# Prosthetic Soft Tissue Management in Esthetic Implant Restorations, Part II: Post‐surgical Considerations and Impression Techniques. A Narrative Review

**DOI:** 10.1002/cre2.70097

**Published:** 2025-03-02

**Authors:** Faezeh Atri, Kimia Nokar

**Affiliations:** ^1^ Department of Prosthodontics School of Dentistry, Craniomaxillofacial Research Center Tehran University of Medical Sciences Tehran Iran; ^2^ Department of Prosthodontics School of Dentistry Tehran University of Medical Sciences Tehran Iran

**Keywords:** dental abutments, dental impression technique, dental prosthesis, implant‐supported, esthetics, dental

## Abstract

**Objectives:**

This two‐part review article delineates various techniques to enhance esthetic outcomes in anterior implant treatments. Part I concentrates on presurgical measures, case selection, implant placement, and restoration timing. Part II discusses postsurgical steps, the temporary restoration phase, the emergence profile contour, abutment types, and impression techniques.

**Material and Methods:**

A comprehensive search was conducted using Medline/PubMed, Embase, Scopus, and the Cochrane Library. The primary keywords included: “Dental Implants,” “Dental Prosthesis, Implant‐Supported,” “Esthetics, Dental,” “Dental Impression Techniques,” and “Tissue Management.”

**Results:**

Initially, 1472 studies were identified, from which 187 were selected based on publication year and title relevance. After removing duplicates, 84 abstracts were reviewed in full text, culminating in 59 studies being thoroughly analyzed.

**Conclusions:**

The decision to deliver an immediate restoration following implant insertion depends on the primary stability. However, in cases where delayed restoration is chosen, it is essential to consider the potential collapse of the soft tissue. A temporary restoration phase could be beneficial to aid in rebuilding the soft tissue. After achieving the desirable level of soft tissue, several techniques are available to achieve a precise transfer of molded gingival architecture, including customized impression coping, injection of soft material around the provisional restoration in the master cast, and digital impression.

## 1 Introduction

Replacing teeth in esthetic zones remains a significant challenge within the clinical setting. In 1982, Dr. Branemark introduced benchmarks for successful dental implants, which included minimal bone loss, optimal gingival health, functionality, and patient satisfaction (Brånemark et al. 1985). By 1989, Smith and Zarb had established criteria for evaluating implant success, notably emphasizing the importance of an adequate esthetic appearance for the first time (Smith and Zarb 1989). Subsequently, in 1996, Garber highlighted that the condition of the peri‐implant soft tissue is a crucial determinant of the esthetic outcome (Garber 1996), and Besler suggested that a successful implant‐supported restoration must replicate the appearance of natural teeth (Belser et al. 2004).

Today, therapeutic success in the anterior region is primarily gauged by function, patient satisfaction, and esthetics. To objectively measure esthetics, indices such as the Pink Esthetic Score and White Esthetic Score have been introduced (Fürhauser et al. 2005). The white esthetic is achieved through effective collaboration between dentists and technicians. In contrast, the pink esthetic assesses the soft tissue surrounding the implant, considering various factors, such as the mesial and distal papilla, soft tissue level, alveolar process deficiency, and the color and texture of the soft tissue (Fürhauser et al. 2005). Creating an appropriate emergence profile and soft tissue contour is predominantly the dentist's responsibility, with the technician needing to adhere to these guidelines during the restoration fabrication.

To achieve desirable esthetic outcomes, clinicians must consider multiple factors, including the gingival phenotype (Lee et al. 2020; Meng et al. 2021; Oh et al. 2006), maintenance of the papilla (Tarnow et al. 1992), the thickness of the buccal bone wall (Monje et al. 2023), implant insertion techniques (Kan et al. 2018), and the timing of implant and restoration procedures (Kinaia et al. 2017; Pitman et al. 2022). This review article aims to discuss the significance of each factor and provide insights that will help clinicians predict the outcomes of treatments.

After implant placement, clinicians have various treatment options to consider. These options may involve delivering either an immediate definitive or temporary restoration, utilizing a customized healing abutment, or a cover screw (Cordaro et al. 2009; de Carvalho Barbara et al. 2019; Greenstein and Cavallaro 2017; Gupta et al. 2019; Meng et al. 2021; Ruales‐Carrera et al. 2019). Once the treatment plan is established, it becomes essential to obtain an accurate impression that captures the gingiva and surrounding structures (Elian et al. 2007). This review article aims to compare different prosthetic treatment options, provide information about potential candidates for each treatment, and outline guidelines for rebuilding collapsed soft tissue. Additionally, it offers an overview of different viable impression‐making techniques.

## 2 Materials and Methods

A search of electronic databases, including Medline/PubMed, Embase, Scopus, and Cochrane, was conducted from January 2008 to May 2023. The following Medical Subject Headings (MeSH) terms and free words were utilized in combinations: (“Dental Implants” OR “Dental Prosthesis, Implant‐Supported”), (“Immediate implant” OR “fresh socket” OR delayed implant), “Immediate Dental Implant Loading,” (“Esthetics, Dental” or “Tissue management”), “Dental Impression Technique.” We included randomized controlled trial studies focusing on implant placement and restoration in the anterior region. Studies involving complex or novel surgical procedures addressed the rehabilitation of completely edentulous patients, bone augmentation or grafting, or utilizing the socket shield technique were excluded. Priority was given to systematic reviews, randomized controlled trials, cohort studies, and clinical guidelines. Only studies published in English were considered.

Patients provided informed consent for the treatment and the use of their photographs in publications.

## 3 Results

Initially, 1472 studies were identified; 187 were selected based on the publication year and title relevance. After the removal of duplicate citations, 84 abstracts of full‐text articles were reviewed, culminating in 59 studies being thoroughly examined and included in the analysis.

### 3.1 Postsurgical Phase

Following implant placement, whether immediate or delayed, it is imperative for clinicians to assess the initial stability of the implant before proceeding to the next phase. Primary stability is a prerequisite for the successful placement of immediate restoration (Gupta et al. 2019; Meng et al. 2021). Research findings indicate that the torque value is a valuable diagnostic tool for evaluating implant stability, with a minimum recommended value of 30–32 Ncm to commence immediate restoration treatment (Gupta et al. 2019; Meng et al. 2021). Non‐occlusive provisional restorations are preferred to maintain the implant undisturbed during the healing phase, and as part of postimplantation care, patients should be instructed to adhere to soft diets and avoid exerting excessive pressure on the restoration (den Hartog et al. 2016; Yan et al. 2016).

However, if primary stability is insufficient, clinicians may choose from other available options, such as placing a definitive abutment, a healing abutment, or a cover screw. The primary objective of these approaches is to preserve the emergence profile. The abutment and provisional restoration can recreate the emergence profile if appropriately shaped.

**Figure 1:**
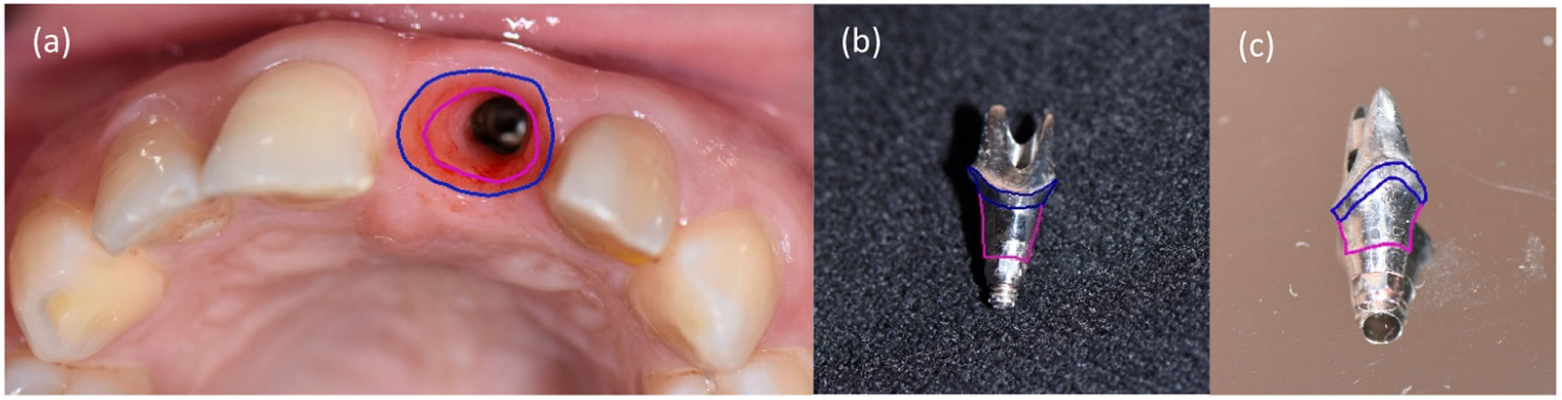
(a) Emergence profile: critical contour (marked in blue), subcritical contour (marked in pink line), (b) Buccal view of the gingival profile of customized abutment. (c) Lateral view of the gingival profile of customized abutment.

The emergence profile comprises two contours:

- Critical contour: Positioned 1 mm below the gingival margin, the critical contour is pivotal in cement‐retained implant restorations. Depending on the placement of the finish line, the critical contour may reside on the crown, abutment, or both. Accurately determining the gingival margin level and zenith position relies heavily on the critical contour (Su et al. 2010). A more bulky critical contour displaces the margin more apically, and altering the critical contour lingually results in coronal migration of the gingival edge (Su et al. 2010).
- Subcritical contour: Situated below the critical contour, the subcritical contour has minimal influence on the gingival margin level but plays a role in tissue support and affects gingival color (González‐Martín et al. 2020; Su et al. 2010). Modifying the subcritical contour, if changing the crown contour is undesired, can enhance the appearance of soft tissue. Sufficient space between the gingival margin and implant neck (“running room”) should be provided by the subcritical contour to facilitate soft tissue formation and prevent tissue compression, thus mitigating the risk of ischemia (González‐Martín et al. 2020). Subsequently, excess soft tissue can be redistributed where needed (Su et al. 2010). It should be noted that with gingival recession, the former subcritical contour assumes the role of the new critical contour (see Figure 1) (González‐Martín et al. 2020).

#### 3.1.1 One Abutment One Time

**Figure 2:**
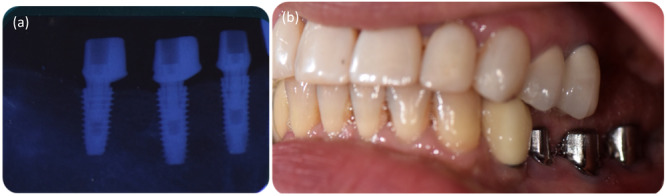
The definitive abutments at the time of implant insertion. (a) Radiographic view, (b) Intraoral view.

The peri‐implant gingival attachment faces disruption due to recurrent abutment detachment (K. Becker et al. 2012; Koutouzis et al. 2013; Tallarico et al. 2018). To mitigate this potential harm, the “one abutment‐one time” protocol has gained recognition recently (de Carvalho Barbara et al. 2019; Fürhauser et al. 2017). This protocol advocates for employing a definitive abutment at the time of implant placement, which remains in situ throughout the entire prosthetic treatment (Canullo et al. 2010; de Carvalho Barbara et al. 2019) (see Figure 2). Research indicates that utilizing the definitive customized abutment from the outset leads to less bone loss than abutment switching, thus offering benefits akin to customized healing abutments (Canullo et al. 2018; de Carvalho Barbara et al. 2019; Esposito et al. 2017; Fürhauser et al. 2017; Molina et al. 2017; Younes et al. 2016). However, the clinical significance of this difference in bone loss is negligible, and both approaches yield comparable esthetic outcomes, making it ultimately the clinician's prerogative to integrate this technique into their treatment plan (Canullo et al. 2010; de Carvalho Barbara et al. 2019; Esposito et al. 2017; Fürhauser et al. 2017).

When considering definitive abutments, hybrid abutments are recommended as a superior alternative to all‐ceramic and titanium abutments. This type of abutment comprises a Computer‐Aided Design/Computer‐Aided Manufacturing (CAD/CAM) milled all‐ceramic mesostructure cemented onto a titanium adhesive base. By amalgamating the strength of titanium with the esthetic appeal of ceramic, this structure proves highly effective, particularly in the anterior region (Edelhoff et al. 2019; Zarauz et al. 2020).

#### 3.1.2 Healing Abutment

Following fresh socket implantation, if a minimum torque of 25 Ncm is attainable (Ruales‐Carrera et al. 2019), a healing abutment can be inserted as an alternative method to support the soft tissue (Akin and Chapple 2022; Chan et al. 2019). A randomized controlled trial (Chan et al. 2019) comparing immediate implantation with prefabricated healing abutment versus immediate provisional restoration found that utilizing a healing abutment yielded similar outcomes regarding mucosal level, papilla height, and overall esthetic results. However, this study evaluated 40 cases, with a 1‐year follow‐up.

Healing abutments can be categorized as either prefabricated or custom‐made via CAD/CAM milling. Nevertheless, prefabricated abutments can also be modified to resemble customized ones. Dental materials such as polyetheretherketone (PEEK), polymethyl methacrylate (PMMA), zirconia, titanium, and resin composite can be used alone or in combination to fabricate customized abutments or modify prefabricated healing abutments (Chokaree et al. 2022).

- *Prefabricated Healing Abutment*
  Conventional healing abutments are available in various diameters and heights but often exhibit a cylindrical cross‐sectional shape, while teeth at the cervical enamel junction are typically triangular or square (W. Becker et al. 2012). This mismatch can lead to technical complications and discomfort during abutment screw tightening and restoration placement (Beretta et al. 2019). At times, the dimensions of conventional healing abutments may not suit a specific case, necessitating modifications by the dentist. Several straightforward chairside approaches can be undertaken without expensive equipment, including modifying prefabricated healing or temporary abutments and utilizing preparable healing abutments. A standard method involves modifying the prefabricated healing abutment by adding composite to achieve the ideal size (see Figure 3).
  **Figure 3:**
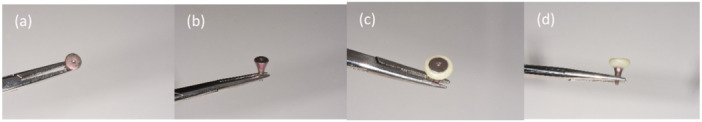
Altering the prefabricated healing abutment with composite resin. (a) Occlusal view of stock gingiva former; (b) Lateral view of stock gingiva former; (c) Occlusal view of altered stock gingiva former, (d) Lateral view of altered stock gingiva former.
  Ruales‐Carrera et al. (2019) delineated steps to place and customize a PEEK prefabricated healing abutment: Initially, the prefabricated healing abutment made of PEEK must be roughened using diamond burs and positioned correctly. Then, the flowable resin should be applied around the healing abutment and light‐cured. Subsequently, the healing abutment can be removed, and critical and subcritical contours must be created using flowable resin and then repositioned. Once finishing and polishing are completed, the customized healing abutment should be stored in 0.12% chlorhexidine until it is screwed onto the implant.
  Studies have demonstrated that using a customized healing abutment, created by adding materials to a prefabricated healing abutment chairside, is a viable approach to maintaining and supporting soft tissue after immediate implants, reducing the number of surgical interventions, and preserving bone substitutes if necessary (Perez et al. 2020; Ruales‐Carrera et al. 2019). This method ensures the emergence profile's preservation for reliable reproduction in the future using either scanning or conventional impression making, as evidenced in a study with 115 cases followed up for up to 8 years (Akin and Chapple 2022).
  Customized healing abutments can be fashioned by adding composite to titanium, or PEEK prefabricated temporary abutment (Akin and Chapple 2022; Ruales‐Carrera et al. 2019). This modified temporary abutment should be reduced to approximately 1 mm above the gingival margin. A case series involving 115 patients with an 8‐year follow‐up utilized this chairside technique in the posterior region, yielding satisfactory ultimate survival and success rates. Moreover, the authors suggested that using a modified healing abutment can preserve the papilla and support the structural integrity of the buccal and lingual walls (Akin and Chapple 2022).
- *Preparable Healing Abutment*
  The concept of preparable healing abutments was pioneered by certain implant manufacturers (Velope et al. 2012). A preparable healing abutment consists of two components with a separate screw and can be shaped according to requirements before being affixed to the fixture. However, these abutments may not always offer an optimal shape for every case and may not be universally available across all implant brands. Consequently, the concept of customized healing abutments has gained traction.
- *CAD/CAM Healing Abutment*
  Customizing a prefabricated healing abutment by adding layers of composite material is a viable method, albeit one that presents challenges and consumes time, particularly when achieving bonding becomes problematic in the presence of bleeding (Beretta et al. 2019; Menchini‐Fabris et al. 2020). Moreover, frequent disconnections of prosthetic components can potentially jeopardize the integrity of the surrounding soft tissues adjacent to the implant (Q. Wang et al. 2017). In contrast, employing a CAD/CAM abutment proves more convenient as it streamlines laboratory procedures, with subgingival contours designed using specialized software (Beretta et al. 2019; Menchini‐Fabris et al. 2020).
  A customized healing abutment surpasses conventional alternatives. Placing a CAD/CAM healing abutment post‐immediate implantation reduces the necessity for surgical interventions and treatment duration, bolsters gingival contour during healing, upholds the emergence profile (Beretta et al. 2019; Menchini‐Fabris et al. 2020; L. Wang et al. 2021), stabilizes bone volume, provides a seal for bone substitute material (Finelle and Lee 2017; L. Wang et al. 2021), and yields a more predictable esthetic outcome for definitive restoration (Beretta et al. 2019; Menchini‐Fabris et al. 2020; L. Wang et al. 2021).
  The procedural steps involved are as follows (Menchini‐Fabris et al. 2020; L. Wang et al. 2021): firstly, obtaining an intraoral scan of the reference tooth (before extraction) (Beretta et al. 2019; Menchini‐Fabris et al. 2020) or the contralateral tooth (Joda et al. 2016; L. Wang et al. 2021). This scan serves as a virtual diagnostic cast for the procedure. In the event of a contralateral tooth scan, mirroring it in software is necessary (L. Wang et al. 2021). After implant placement, a second scan is conducted using a scan body to ensure precise transfer of the implant position. The virtual crown is then designed and truncated above the gingival margin to encompass solely the requisite emergence profile. It is pertinent to mention that the emergence profile can be designed with either the same diameter or a 10% reduction to accommodate future inflammation and forestall gingival recession (L. Wang et al. 2021). Once the design phase is concluded, the final healing abutment can be milled from materials such as Titanium, PEEK (35), or PMMA blocks (L. Wang et al. 2021). Optionally, the emergence profile portion can be milled separately and integrated with a prefabricated component, if necessary. Subsequently, the abutment is securely affixed to the implant, culminating the process with assurance. It is advisable to wait a period of 6 to 8 weeks before proceeding with the final impression (Proussaefs 2016).

#### 3.1.3 Cover Screw

An alternative approach is to employ a cover screw. From an esthetic standpoint, suturing the surrounding soft tissue of the cover screw in a non‐submerged implant protocol is marginally preferable to a submerged one (Cordaro et al. 2009). Following the healing phase, delayed restorative treatment is administered to the patient. Given significant soft tissue collapse, a gradual, step‐by‐step provisional phase is recommended to restore the tissue and interdental papilla effectively. This provisional phase entails manipulating the emergence profile of an interim restoration over multiple appointments, thereby guiding the tissue toward achieving ideal scalloping before the final impression is taken. The provisional phase has proven highly advantageous for optimal tissue regeneration (Furze et al. 2019; Sutariya et al. 2022).

### 3.2 Contour Management in the Provisional Phase

González‐Martin et al. (2020) proposed guidelines for manipulating provisional restorations, detailed below:

For immediate provisional restoration, the primary objective is to support and preserve soft tissue. This can be achieved by employing a critical contour with a reduction of 0.5–1 mm on the gingival aspect and dimensions equivalent to those of the natural tooth on the interproximal and palatal aspects, complemented by a subcritical contour that is concave on all aspects.

In the case of delayed provisional restoration, the principal aim is to recreate an appropriate emergence profile and gingival margin. Actions can be taken to achieve the desired gingival margin based on the soft tissue level present. An over‐dimensioned critical contour with a flat or slightly concave subcritical contour may lead to facial tissue recession, whereas an under‐dimensioned critical contour with a more convex subcritical contour can induce gingival tissue coronal migration. If the existing soft tissue level is optimal, the critical contour should mirror that of a natural tooth, while the subcritical contour should be flat or slightly under‐contoured. Once the desired mucosal level is reached, the subcritical contour should be further under‐contoured to facilitate additional tissue regeneration, resulting in thicker soft tissue.

A recent randomized controlled trial (Siegenthaler et al. 2022), comprising 84 cases with a 12‐month follow‐up, assessed the impact of emergence profile (convex or concave) on gingival margin. As anticipated, they concluded that employing a temporary restoration with an under‐contoured or concave emergence profile leads to more stable marginal levels. Regarding the abutment emergence profile, satisfactory esthetic outcomes were demonstrated by Schoenbaum et al. (2013) after recontouring a flared stock‐shaped titanium provisional abutment into a parallel one. Hence, a straight or concave design is recommended. Such a design facilitates soft tissue generation, alleviates pressure on surrounding tissue, and diminishes bone resorption (Otero et al. 2018; Schoenbaum et al. 2013).

**Figure 4:**
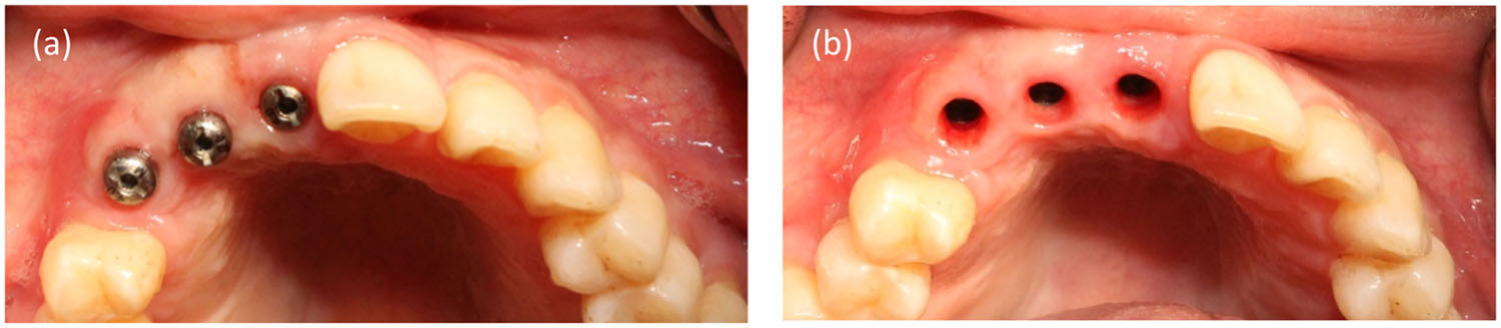
Intraoral view showing healed gingiva with inappropriate shape: (a) Thin stock healing abutment, (b) Undersized emergence profile.

A clinical case illustrating tissue management in delayed restoration is presented. A woman with three implants was referred to a prosthodontist for restoration fabrication. The healed gingival shape did not meet ideal esthetic standards. The gingival margin and diameter were asymmetrical compared to contralateral reference teeth, necessitating soft tissue molding through provisional restorations (Figure 4).

**Figure 5:**
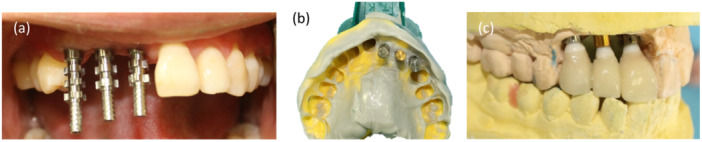
Fabrication process of the primary provisional restoration: (a) Stock open impression coping; (b) Conventional impression making; (c) Laboratory‐processed screw‐type provisional restorations.

**Figure 6:**
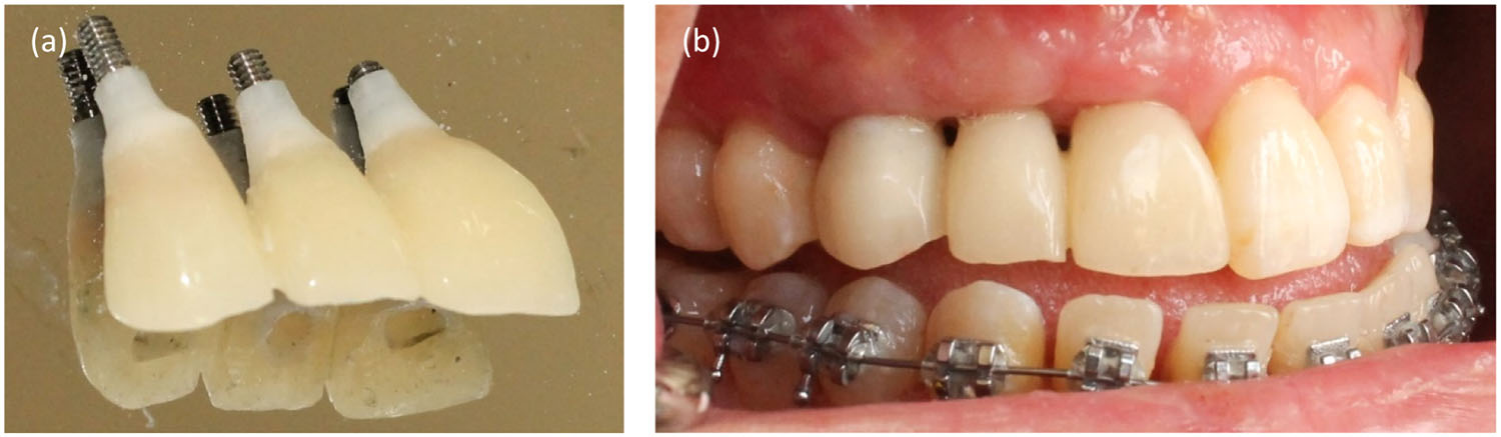
(a) Laboratory‐processed screw‐type provisional restorations with undersized gingival profile, (b) Delivery of the primary provisional restoration, highlighting the absence of papillae.

In the initial step, impressions were taken to fabricate a provisional restoration (Figure 5). The provisional was subsequently delivered, with the emergence profile of the primary provisional being narrower and the papillae space left unfilled (Figure 6).

**Figure 7:**
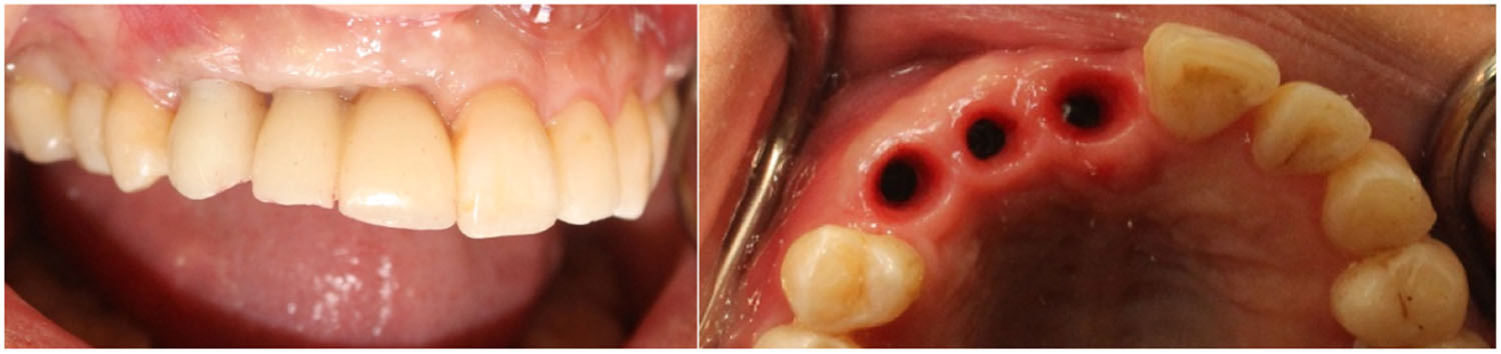
(a) Papillae formation around the provisional after 3 weeks; (b) Formed gingival emergence profile.

After 3 weeks, the patient was reassessed. The gingiva was molded around the restoration, filling the papillae and resulting in a broader emergence profile (Figure 7).

Subsequently, the provisional was removed from the mouth, and a critical contour was added with composite material to displace the margin more apically and gradually widen the emergence, thereby achieving a mature and natural shape ready for the final restoration.

### 3.3 Accurate Transfer of Peri‐Implant Soft Tissue

Conventional stock impression copings possess a uniform round shape and, consequently, cannot accurately replicate anatomical variations and the scalloped shape of the gingiva (Elian et al. 2007; Velásquez et al. 2015). The gingival shape lacks support during conventional impression‐making, leading to immediate collapse. Consequently, transferring the soft tissue shape to the dental technician becomes challenging. In such scenarios, the technician may create an emergence profile that does not align with the morphology of the existing soft tissue (Elian et al. 2007), potentially causing discomfort for the patient during insertion. To circumvent these issues, the final prosthetic crown must mirror any intraoral soft tissue alterations made by the temporary restoration (Elian et al. 2007). Several techniques have been proposed in the literature for precise communication of peri‐implant soft tissue to technicians, with four methods gaining popularity:

(1) *Customized Impression Coping*

A customized impression coping tailored to the pre‐implant soft tissue profile can be fabricated using direct or indirect techniques. A study by Velásquez et al. (2015) demonstrated that the indirect method more accurately captures all three implant‐supported fixed temporary restoration levels—platform, middle, and gingival margin than the direct method. However, the direct technique is accurate only at the platform level.

In the direct technique (Velásquez et al. 2015), a conventional impression coping is modified intraorally by adding composite or acrylic to support the gingival shape. This modification should be performed swiftly before tissue collapse occurs. Once the provisional is removed, the coping is screwed in place, and composite or acrylic is injected around it to record the soft tissue scallop. An extraoral modification of the coping from an index derived from the provisional restoration is advisable for cases involving multiple implants.

In the indirect technique (Shor et al. 2008; Vasconcellos and Proussaefs 2016; Velásquez et al. 2015), the provisional restoration is removed from the mouth and attached to the implant analog. A putty silicone index is then made from the gingival part of the restoration. Subsequently, the provisional is detached from the analog, and the impression coping is attached. The gap between the silicone index and coping is filled with composite or acrylic to replicate the patient's gingival contour.

(2) *Emergence Profile Cast* (Attard and Barzilay 2003; Elian et al. 2007; Tsai 2011)

The provisional restoration can serve as an impression coping in this method. Long impression screws are used to make an open tray or a pick‐up impression. A conventional closed‐tray impression can also be taken with the provisional restoration. Subsequently, the provisional is removed, attached to the analog, and transferred to the tray. However, this method has two drawbacks despite its accuracy (Attard and Barzilay 2003; Elian et al. 2007): the patient does not receive a temporary restoration until the master cast is produced, and the process is time‐consuming (Velásquez et al. 2015).

(3) *Injection of Soft Material Around Provisional Restoration in the Master Cast* (Yilmaz 2015)

Conventional impression‐making is conducted, and a master cast is prepared using this technique. The soft silicone around the analog is removed, and the exact level of the gingiva is marked on the provisional restoration. The provisional is then removed and transferred to the master cast. The soft silicone material is injected around the provisional to reproduce the gingival contour. This chairside technique allows the patient to retain the temporary restoration while the permanent restoration is fabricated, eliminating the need for a second impression. However, adjustments to the substitute for soft tissue may be necessary (Alani and Corson 2011).

(4) *Digital Impression*

In contemporary dentistry, intraoral scanners have become reliable tools for accurately capturing soft tissue surrounding dental implants (Marques et al. 2021; Michelinakis et al. 2021). Digital technology reduces chair time and the number of laboratory procedures while delivering predictable, definitive restorations (Monaco et al. 2019). However, the accuracy of digital impressions may be affected by factors, such as the impression‐taking protocol and implant depth (Marques et al. 2021).

Various techniques can be employed with digital technology in impression‐taking. One such technique involves integrating multiple images. Monaco et al. (2019) introduced direct and indirect protocols for digital impression‐taking based on emergence profile shape and implant depth.

**Figure 8:**
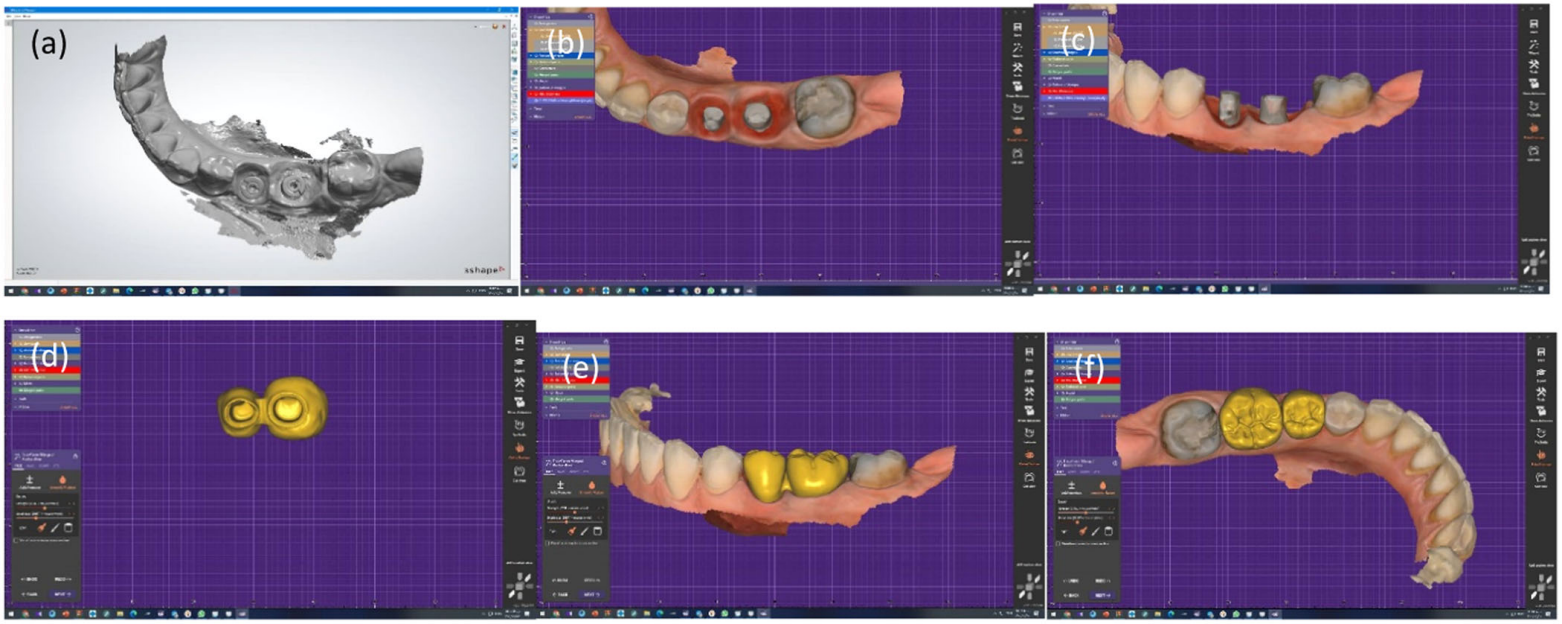
Digital impression of emergence profile using the direct method: (a) Intraoral scan; (b and c) Customized abutment design; (d) Gingival profile of the final restoration; (e and f) Appropriate gingival contour.

The direct method requires stability of peri‐implant tissues within 1 min after removing the provisional restoration. It is indicated for cases with cone‐shaped emergence profiles and short, thick peri‐implant soft tissue, where the depth of the implant platform from peri‐implant soft tissue is less than 4 mm. Three digital impressions are taken: a full arch scan with the scan body in place serves as a reference impression, supplemented by two additional impressions—one with the provisional restoration in place and another within 10 s after removing the provisional restoration (Monaco et al. 2019) (Figure 8).

**Figure 9:**
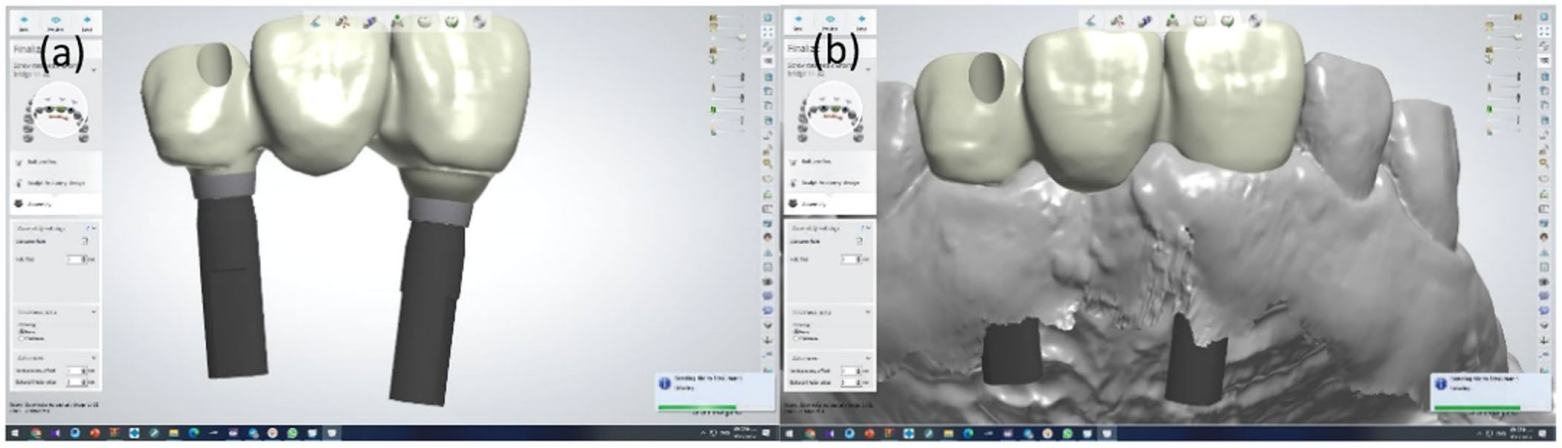
Digital impression of emergence profile using the indirect method: (a) Scan of the provisional restoration on an implant analog outside the mouth, (b) Superimposition of the scanned provisional restoration on the oral scan.

The indirect method is indicated when soft tissue stability is lacking, the emergence profile is cylindrical, and the depth exceeds 4 mm. In this case, three digital impressions are taken: a full arch scan with a scan body in place as the reference impression, supplemented by one with the provisional restoration in place and another with the provisional restoration on an implant analog outside the mouth (Monaco et al. 2019) (Figure 9).

## 4 Conclusions

Achieving ideal esthetics in implant restorations requires precise treatment planning, proper surgical technique, and meticulous steps in the restorative process to ensure predictable outcomes. Once the implant is placed, various options, such as provisional restorations, definitive abutments, temporary abutments, or cover screws, may be used based on primary stability and clinical judgment. The emergence profile should be shaped to match the presurgical contours if an abutment is chosen. Utilizing CAD/CAM customized abutments or manipulating prefabricated abutments to simulate natural contours is recommended. Alternatively, if a cover screw is selected, the emergence profile of the provisional restoration should be gradually adjusted to rebuild the proper gingival contour. Once the gingiva is properly contoured, impression‐taking can be accomplished using four possible techniques: (1) customized impression coping, (2) emergence profile cast, (3) injection of soft material around the provisional restoration in the master cast, and (4) digital impression. The clinician must select the appropriate technique based on the patient's condition, needs, and available materials and equipment.

## Author Contributions

Conceptualization: Faezeh Atri. Methodology: Faezeh Atri and Kimia Nokar. Search and Selection: Kimia Nokar. Writing – original draft preparation: Faezeh Atri and Kimia Nokar. Writing – review and editing: Faezeh Atri and Kimia Nokar. Critical review: Faezeh Atri. All authors read and approved the final manuscript.

## Conflicts of Interest

The authors declare no conflicts of interest.

## Data Availability

The authors have nothing to report.
